# Evaluation of Identification and Susceptibility for *Candida* Spp. Isolated Directly from Positive Blood Culture Bottles

**DOI:** 10.1155/2021/9364231

**Published:** 2021-11-16

**Authors:** Ândrea Celestino de Souza, Luciano Z. Goldani, Eliane Würdig Roesch, Larissa Lutz, Patricia Orlandi Barth, Paulo André de Souza Sampaio, Valério Rodrigues Aquino, Dariane Castro Pereira

**Affiliations:** ^1^Microbiology Unit, Hospital de Clínicas de Porto Alegre, Universidade Federal Do Rio Grande do Sul, Porto Alegre, Brazil; ^2^Infectious Diseases Section, Universidade Federal Do Rio Grande do Sul, Porto Alegre, Brazil

## Abstract

Determination of the susceptibility profile of isolates of *Candida* from blood culture bottles is extremely important for correctly guiding patient pharmacotherapy. The aim of this study was to compare the results of analysis of *Candida* isolated directly from blood culture bottles by the VITEK MS MALDI-TOF identification system and the fluconazole disk diffusion assay with those of standard identification methods. Testing directly from the bottle allowed results 24 to 48 hours quicker than the standard method. There was a categorical agreement of 51.64% (47 of 91 samples) between the results of analysis directly from the bottle and analysis by the standard method. Regarding species identification, there was 96.15% agreement for *Candida parapsilosis* (25 of 26 samples). Categorical agreement between the rapid and standard disk diffusion methods was 95%, and the agreement between the rapid disk diffusion method and the broth microdilution method was 97%. Only minor errors in the rapid method were observed: 3 (5%) in the standard disk diffusion method and 2 (3%) in the broth microdilution method. Our study concluded that the rapid disk diffusion method for fluconazole is a fast, easy, reproducible, and consistent method. Its timely implementation for testing antifungal agents in the clinical microbiology laboratory can help reduce profile release times, thus helping to determine the most appropriate antifungal treatment.

## 1. Introduction

Bloodstream infections caused by *Candida* species are becoming increasingly common in hospitals, with the incidence being fivefold higher than that in the last decade, and are associated with high morbidity and mortality rates [1, 2]. Fluconazole is a well-tolerated triazole antifungal with high bioavailability and tissue penetration ability [3]. However, prolonged treatment could induce resistant mutations in *Candida* that lead to therapeutic failure, which is a critical concern since fluconazole is the most commonly used antifungal agent for the prophylaxis and treatment of *Candida* infections in many parts of the world [1].

In a 9-year retrospective cohort study at a 1250-bed US Hospital, Kollef et al. found that the hospital mortality rate for septic shock patients who received antifungal therapy within 24 hours of candidemia onset was 52.8% (*n* = 142), compared to 97.6% (*n* = 82) in those who did not receive antifungal therapy [4]. Other studies have found that the 30-day survival rate of candidemia patients who receive appropriate pharmacotherapy was better than that of patients who received delayed or no treatment. These studies show that late initiation of adequate pharmacotherapy in infected patients correlates with an increased mortality rate [5–7]. Thus, determining the species responsible for the infection and the susceptibility profile of *Candida* spp. is important not only for guiding pharmacotherapy but also for monitoring the treatment efficacy and the emergence of resistance.

This study aimed to use a rapid method to identify species of *Candida* and assess fluconazole susceptibility directly from positive blood cultures.

## 2. Methods

The study was approved by the local ethics committee.

### 2.1. Yeast Strains

Isolates of *Candida* were obtained from blood cultures of patients admitted to a tertiary care hospital in Southern Brazil. For the rapid identification method, we included all the samples of blood cultures from patients with *Candida* species isolated between September 2018 and June 2019. For the rapid disk diffusion method, we included only one sample per patient between September 2018 and September 2019.

Blood samples were inoculated in aerobic bottles and incubated in the BacT/ALERT^®^ 3D system (bioMérieux, France) for microorganism growth monitoring. We excluded from the study samples from which more than one microbial species was isolated. *Candida albicans* ATCC 90028, *Candida tropicalis* ATCC 750, *Candida krusei* ATCC 6258, and *Candida parapsilosis* ATCC 90018 were included as quality control strains.

### 2.2. Standard Method

Following microorganism growth identification by the BacT/ALERT^®^ 3D system, blood culture media were collected from each bottle and subjected to Gram staining. Then, samples were subcultured on solid growth media, including blood agar (bioMérieux) and Sabouraud agar (Merck, Germany), and incubated at 35°C for 18–24 hours. To estimate the cell numbers in the bottles, 5 positive blood culture bottles were randomly selected. Then, 1 mL sample was aspirated from each of these bottles, serially 10‐fold diluted with sterile saline, and 50 *μ*L of suspensions was plotted on the Sabouraud agar plate, and colonies were counted after 24 h of incubation (ranged from 7 × 10^5^ to 5 × 10^7^ CFU/mL). The rapid disc diffusion method was performed according to the RAST methodology standardized by the European Committee on Antimicrobial Susceptibility Testing. Following incubation, isolated colonies were subjected to analysis by the MALDI-TOF VITEK MS^®^ 3.0 system (bioMérieux, France) according to the manufacturer's instructions. Fluconazole susceptibility was assessed using a disk diffusion method according to the CLSI M44-A2 guidelines and a broth microdilution method according to the European Committee on Antimicrobial Susceptibility Testing guidelines [8, 9].

### 2.3. Rapid Identification Method

The rapid identification method was performed according to the protocol proposed by Spanu et al. [10]. Each test was conducted in duplicate. An 8 ml aliquot from the blood culture bottle was centrifuged at 10,000 rpm for 2 minutes at room temperature. The supernatant was discarded, and the pellet was washed twice with 1 ml of pure water and recentrifuged. It was suspended in 1 ml of 0.1% Tween 80, incubated for 2 minutes, recentrifuged, washed twice with 1 ml of pure water, recentrifuged, suspended in 300 *μ*l of pure water plus 900 *μ*l of absolute ethanol, and recentrifuged. Then, 30 *μ*l of 70% formic acid plus 30 *μ*l of pure acetonitrile was added to the pellet, and it was thoroughly vortexed and centrifuged at 14,000 rpm for 2 minutes. A 1 *μ*l aliquot of the supernatant was collected and applied to a steel MALDI target plate. Finally, the sample was subjected to analysis by the MALDI-TOF VITEK MS^®^ 3.0 system (bioMérieux, France).

### 2.4. Rapid Disk Diffusion Method

The rapid disk diffusion method was performed according to Jabeen et al. [11]. A 100 *μ*L aliquot from the blood culture bottles was used to make lawns on Mueller–Hinton agar supplemented with 2% dextrose and 0.5 *μ*g/ml methylene blue dye. Two discs with 25 *μ*g of fluconazole were placed on the plates, and the plates were incubated.

### 2.5. Statistical Analysis

The kappa coefficients and categorical agreement of the data were determined using software PASW v.18 (IBM, USA). The acceptable rate agreement was ≤90% (10). Errors were classified into very major errors, major errors, and minor errors, and the acceptable rates were ≤1.5%, ≤3%, and ≤10%, respectively [12].

## 3. Results

A total of 91 blood culture samples from 46 patients were tested by rapid identification method tests. The overall agreement of *Candida* species identification between the rapid and standard methods was 51.64%. *Candida parapsilosis* had the highest agreement (96.15%) of the tested samples (Table 1). No sample containing *Candida orthopsilosis* or *Candida pelliculosa* was identified, and the agreement for other species varied from 30 to 67% (Table 1).

A total of 62 samples were used to assess fluconazole susceptibility by the standard disk diffusion method, the rapid disk diffusion method, and the broth microdilution method.  Table 2 presents the susceptibility profiles of the *Candida* species isolated in the study obtained by the gold standard method (broth microdilution). The minimum inhibitory concentration (MIC) found for *Candida* spp. ranged from 0.125 to 32.0 *μ*g/mL, and MIC 50 and MIC 90 were 0.5 and 4.0 *μ*g/mL, respectively. For *Candida albicans,* the MIC range was 0.125 to 1.0 *μ*g/mL, and MIC 50 and MIC 90 were 0.5 *μ*g/mL and 1.0 *μ*g/mL, respectively. For the *Candida parapsilosis* complex, the MIC range was 0.25 to 4.0 *μ*g/mL, and MIC 50 and MIC 90 were 1.0 and 2.0 *μ*g/mL, respectively. Approximately 87% of *Candida* spp. samples were sensitive to fluconazole (all *C. albicans* and 80% of non-*albicans Candida* isolates).

**Figure 1:**
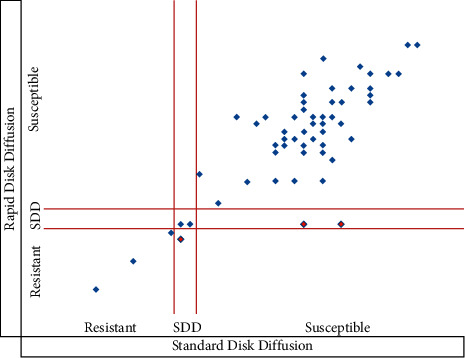
Distribution of susceptible profiles of *Candida* spp. according to the standard and rapid disk diffusion methods. SDD: fluconazole susceptible-dose dependent.

**Figure 2:**
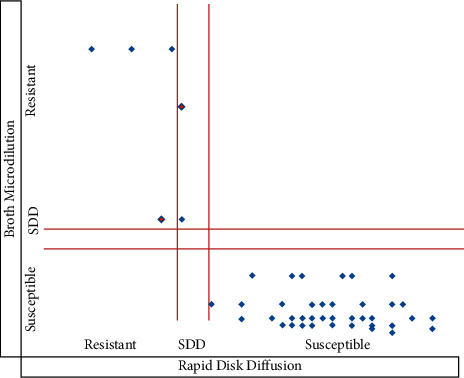
Distribution of susceptible profiles of *Candida* spp. according to the broth microdilution method and the rapid disk diffusion method. SDD: fluconazole susceptible-dose dependent.

The categorical agreement between the rapid disk diffusion method and the standard disk diffusion method was approximately 95% and involved 3 minor errors (5%) (Figure 1). The kappa coefficient (*K* = 0.77; *p* < 0.001) showed strong agreement between these two methods. The categorical agreement between the rapid disk diffusion method and the broth microdilution method was 97% and involved 2 minor errors (3%) (Figure 2). The kappa coefficient (*K* = 0.86; *p* < 0.001) showed almost perfect agreement between these methods. A comparison of the results of the rapid disk diffusion method, the standard disk diffusion method, and the broth microdilution method (gold standard) is shown in Figures 1 and 2.

## 4. Discussion

The classical diagnostic workflow takes up to several days due to the slow growth of yeasts. The overall performance of our standard identification (Bruker Biotyper and VITEK MS) was in accordance with published data, with 70.7% of yeast correctly identified to the species, genus, or complex level [12]. Unlike in the study conducted by Lévesque et al., the overall identification rate for our rapid method was lower than expected (52%) [13]. These authors used the Bruker Biotyper MALDI-TOF system (Daltonik GmbH, Leipzig, Germany) and obtained identification rates of 95.9% for *C. albicans* and 86.5% for non-*albicans Candida* species.

The ability to rapidly identify *Candida* species may be useful to promptly streamline the development of antifungal therapy based on empirical evidence [14, 15]. However, the emergence and spread of fluconazole-resistant *Candida* have introduced a pressing need for rapid antifungal susceptibility tests [16]. Our rapid disk diffusion method was reproducible, yielding concordant results and few errors compared to standard disk diffusion and broth microdilution methods. For our method, there were three minor errors compared to the standard disk diffusion method for the *C. parapsilosis* complex, two minor errors compared to broth microdilution for *C. glabrata* and *C. parapsilosis*, no errors for *C. albicans* isolates (which have the highest incidence in the hospital, 35%), and no errors for *C. krusei* and *C. tropicalis*. Moreover, in this study, the rapid disk diffusion method was more reliable for broth microdilution, which is the gold standard, than for standard disk diffusion, exhibiting a smaller number of errors, a higher kappa, and a higher categorical agreement rate.

**Figure 3:**
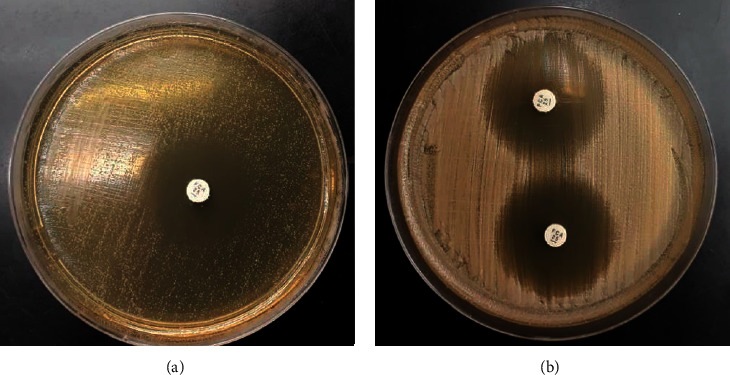
Comparison between standard disk diffusion (a) and rapid disk diffusion (b) susceptibility testing of *Candida* spp. for fluconazole.

The results indicate that the rapid disk diffusion test is promising for testing additional antifungal agents in microbiology laboratories, given that it can shorten the time needed for the identification of *Candida* spp. susceptibility profiles by up to two days [17]. This direct method saved on average 21.5 h for identification and 12.1 h for susceptibility testing compared to standard methods. The test is practical, easy to use, inexpensive, and rapid. It eliminates process steps, and interpreting halos is clearer and safer. Therefore, with this method, the halo is better delimited, which prevents conflicting results and interoperator error, as shown in Figure 3. By releasing susceptibility profile results more quickly, harm from inappropriate and sometimes ineffective pharmacotherapy can be reduced, aiding in patient recovery and reducing mortality and the length of stay, thus contributing to better patient safety. Further studies of susceptibility testing for other antifungal agents including echinocandins are necessary.

## Figures and Tables

**Table 1:** *Candida* species identified directly from 91 blood culture samples from 46 patients.

Standard identification	Number of isolates		% agreement
	Total tested	Identification matching	
*Candida albicans*	28	14	50.00
*Candida glabrata*	3	2	66.67
*Candida krusei*	5	3	60.00
*Candida orthopsilosis*	16	0	0
*Candida parapsilosis*	26	25	96.15
*Candida pelliculosa*	3	0	0
*Candida tropicalis*	10	3	30.00
Total	91	47	51.64

**Table 2:** Distribution of *Candida* spp. and their fluconazole susceptibility profiles according to the gold standard (broth microdilution). The minimum inhibitory concentration (MIC) found for *Candida* spp. ranged from 0.125 to 32.0 *μ*g/mL, and MIC 50 and MIC 90 were 0.5 and 4.0 *μ*g/mL, respectively.

Species	Sensitive isolates	Dose-dependent isolates	Resistant isolates	Total isolates
*Candida albicans*	22	0	0	22
*Candida dubliniensis*	1	0	0	1
*Candida glabrata*	0	0	2	2
*Candida krusei*	0	0	2	2
*Candida orthopsilosis*	9	2	0	11
*Candida parapsilosis*	15	1	0	16
*Candida pelliculosa*	1	0	0	1
*Candida tropicalis*	6	1	0	7
Total	54	4	4	62

## Data Availability

The data used to support the findings of this study are included within the article.

## References

[1] Bassetti M., Merelli M., Righi E. (2013). Epidemiology, species distribution, antifungal susceptibility, and outcome of candidemia across five sites in Italy and Spain. *Journal of Clinical Microbiology*.

[2] Lortholary O., Renaudat C., Renaudat C. (2014). Worrisome trends in incidence and mortality of candidemia in intensive care units (Paris area, 2002–2010). *Intensive Care Medicine*.

[3] Peron I. H., Reichert-Lima F., Busso-Lopes A. F. (2016). Resistance surveillance in Candida albicans: a five-year antifungal susceptibility evaluation in a Brazilian university hospital. *PLoS One*.

[4] Kollef M., Micek S., Hampton N., Doherty J. A., Kumar A. (2012). Septic shock attributed to Candida infection: importance of empiric therapy and source control. *Clinical Infectious Diseases*.

[5] Cortés J. A., Reyes P., Gómez C. H. (2014). Clinical and epidemiological characteristics and risk factors for mortality in patients with candidemia in hospitals from Bogotá, Colombia. *Brazilian Journal of Infectious Diseases*.

[6] Ghanem-Zoubi N., Zorbavel D., Khoury J. (2018). The association between treatment appropriateness according to EUCAST and CLSI breakpoints and mortality among patients with candidemia: a retrospective observational study. *European Journal of Clinical Microbiology & Infectious Diseases*.

[7] Puig-Asensio M., Pemán J., Zaragoza R. (2014). Impact of therapeutic strategies on the prognosis of candidemia in the ICU. *Critical Care Medicine*.

[8] CLSI (2009). *M44-A2: Method for Antifungal Disk Diffusion Susceptibility Testing of Yeasts; Approved Guideline—Second Edition*.

[9] European Committee on Antimicrobial Susceptibility Testing (2020). *Antifungal Agents-Breakpoint Tables for Interpretation of MICs*.

[10] Spanu T., Posteraro B., Fiori B. (2012). Direct maldi-tof mass spectrometry assay of blood culture broths for rapid identification of Candida species causing bloodstream infections: an observational study in two large microbiology laboratories. *Journal of Clinical Microbiology*.

[11] Jabeen K., Kumar H., Farooqi J., Mehboob R., Brandt M. E., Zafar A. (2016). Agreement of direct antifungal susceptibility testing from positive blood culture bottles with the conventional method for Candida species. *Journal of Clinical Microbiology*.

[12] International Organization for Standardization (ISO) (2007). *Clinical Laboratory Testing and In Vitro Diagnostic Test Systems. Susceptibility Testing of Infectious Agents and Evaluation of Performance of Antimicrobial Susceptibility Test Devices. Part 2: Evaluation of Performance of Antimicrobial*.

[13] Lévesque S., Dufresne P. J., Soualhine H. (2015). Lefebvre B, a side by side comparison of bruker biotyper and VITEK MS: utility of MALDI-TOF MS technology for microorganism identification in a public health reference laboratory. *PLoS One*.

[14] Idelevich E. A., Grünastel B., Becker K. (2017). Rapid detection and identification of candidemia by direct blood culturing on solid medium by use of lysis-centrifugation method combined with matrix-assisted laser desorption ionization-time of flight mass spectrometry (MALDI-TOF MS). *Journal of Clinical Microbiology*.

[15] Marinach-Patrice C., Fekkar A., Atanasova R. (2010). Rapid species diagnosis for invasive candidiasis using mass spectrometry. *PLoS One*.

[16] Pfaller M. A., Diekema D. J. (2007). Epidemiology of invasive candidiasis: a persistent public health problem. *Clinical Microbiology Reviews*.

[17] Khan Z. A., Siddiqui M. F., Park S. (2019). Current and emerging methods of antibiotic susceptibility testing. *Diagnostics*.

